# Cerebellum to motor cortex paired associative stimulation induces bidirectional STDP-like plasticity in human motor cortex

**DOI:** 10.3389/fnhum.2012.00260

**Published:** 2012-09-19

**Authors:** Ming-Kuei Lu, Chon-Haw Tsai, Ulf Ziemann

**Affiliations:** ^1^Department of Neurology, Goethe-UniversityFrankfurt/Main, Germany; ^2^Neuroscience Laboratory, Department of Neurology, China Medical University HospitalTaichung, Taiwan; ^3^Institute of Medical Science and School of Medicine, China Medical UniversityTaichung, Taiwan; ^4^Department of Neurology, Hertie-Institute for Clinical Brain Research, University Hospital TübingenTübingen, Germany

**Keywords:** cerebello-dentato-thalamo-cortical pathway, spike-timing dependent-like plasticity, cerebellum, motor cortex, transcranial magnetic stimulation, paired associative stimulation, human

## Abstract

The cerebellum is crucially important for motor control and adaptation. Recent non-invasive brain stimulation studies have indicated the possibility to alter the excitability of the cerebellum and its projections to the contralateral motor cortex, with behavioral consequences on motor control and adaptation. Here we sought to induce bidirectional spike-timing dependent plasticity (STDP)-like modifications of motor cortex (M1) excitability by application of paired associative stimulation (PAS) in healthy subjects. Conditioning stimulation over the right lateral cerebellum (CB) preceded focal transcranial magnetic stimulation (TMS) of the left M1 hand area at an interstimulus interval of 2 ms (CB→M1 PAS_2 ms_), 6 ms (CB→M1 PAS_6 ms_) or 10 ms (CB→M1 PAS_10 ms_) or randomly alternating intervals of 2 and 10 ms (CB→M1 PAS_Control_). Effects of PAS on M1 excitability were assessed by the motor-evoked potential (MEP) amplitude, short-interval intracortical inhibition (SICI), intracortical facilitation (ICF) and cerebellar-motor cortex inhibition (CBI) in the first dorsal interosseous muscle of the right hand. CB→M1 PAS_2 ms_ resulted in MEP potentiation, CB→M1 PAS_6 ms_ and CB→M1 PAS_10 ms_ in MEP depression, and CB→M1 PAS_Control_ in no change. The MEP changes lasted for 30–60 min after PAS. SICI and CBI decreased non-specifically after all PAS protocols, while ICF remained unaltered. The physiological mechanisms underlying these MEP changes are carefully discussed. Findings support the notion of bidirectional STDP-like plasticity in M1 mediated by associative stimulation of the cerebello-dentato-thalamo-cortical pathway and M1. Future studies may investigate the behavioral significance of this plasticity.

## Introduction

The cerebellum is essentially important for control of posture and movement (Brooks and Thach, 1981) and for specific motor learning processes, in particular motor adaptation (Shmuelof and Krakauer, 2011). The cerebellum has abundant efferent projections to distributed areas of the sensorimotor cortex (Allen and Tsukahara, 1974; Thach, 1987; Middleton and Strick, 1998; Hoover and Strick, 1999) and influences motor behavior through these connections. Excitability of the cerebellum and its cerebello-dentato-thalamo-cortical connections can be altered in humans by non-invasive brain stimulation techniques such as transcranial magnetic stimulation (TMS) and transcranial direct current stimulation of the lateral cerebellum (Oliveri et al., 2005; Fierro et al., 2007; Koch et al., 2008; Galea et al., 2009, 2011; Popa et al., 2009). Concomitantly, non-invasive stimulation of the lateral cerebellum can lead to changes in the spatial and timing precision of hand movements (Miall and Christensen, 2004; Del Olmo et al., 2007), adaptive motor learning (Galea et al., 2011; Panouilleres et al., 2012), associative motor learning (Hoffland et al., 2012), and procedural motor learning (Torriero et al., 2004).

Paired associative stimulation (PAS) is a well explored stimulation technique that allows induction of bidirectional spike-timing dependent plasticity (STDP)-like plasticity (for reviews, Ziemann et al., 2008; Müller-Dahlhaus et al., 2010). Depending on the interstimulus interval between an afferent input into the primary motor cortex (M1) and action potential generation in M1 corticospinal neurons by suprathreshold TMS, long-term depression (LTD)-like or long-term potentiation (LTP)-like plasticity of corticospinal neurons occurs, strongly reminiscent of STDP as studied at the level of single cells in brain slices or neuronal cultures (Markram et al., 1997; Bi and Poo, 2001). Bidirectional STDP-like plasticity has so far been demonstrated for repeated pairing of TMS of M1 with afferent inputs into M1 from peripheral nerves (Stefan et al., 2000, 2002; Wolters et al., 2003; Ziemann et al., 2004; Müller et al., 2007) and from the ipsilateral ventral premotor cortex (Buch et al., 2011).

Here we sought to test the possibility to induce STDP-like plasticity along the cerebellar-dentato-thalamo-M1 connection by cerebellum-to-M1 (CB→M1) PAS. In doing so, several peculiarities of this pathway have to be taken into consideration: TMS of the lateral cerebellum most likely excites Purkinje cells (Ugawa et al., 1995), i.e., the principal inhibitory neurons of the cerebellum. This leads to inhibition of target neurons in the dentate nucleus and, consequently, to disfacilitation of the tonically active bi-synaptic excitatory projection to M1, measurable as “cerebellar-motor inhibition” (CBI) of motor-evoked potentials (MEPs) (Ugawa et al., 1995).

We show that CB→M1 PAS leads to MEP depression or MEP potentiation, depending on the interstimulus interval between the CB and M1 stimuli, most likely explained by Hebbian weakening or strengthening, respectively, of the cerebello-dentato-thalamo-M1 connection. This bidirectional modification of M1 excitability may prove useful for correcting abnormal M1 excitability caused by cerebellar disease (Groiss and Ugawa, 2012). Also, behavioral effects of CB→M1 PAS, in particular on motor performance and various forms of motor learning are possible and warrant further investigation.

## Methods

### Subjects

Nineteen healthy subjects participated in this study (mean age ± SD, 29.8 ± 6.9 years; range, 22–42 years; four female). All subjects were right-handed according to the Edinburgh Handedness Inventory (Oldfield, 1971). All gave their written informed consent prior to the study. The experimental procedures were in accord with the Declaration of Helsinki and approved by the local Ethics Committee of the medical faculty of the Goethe-University of Frankfurt am Main, Germany.

### Experimental design

**Figure 1:**
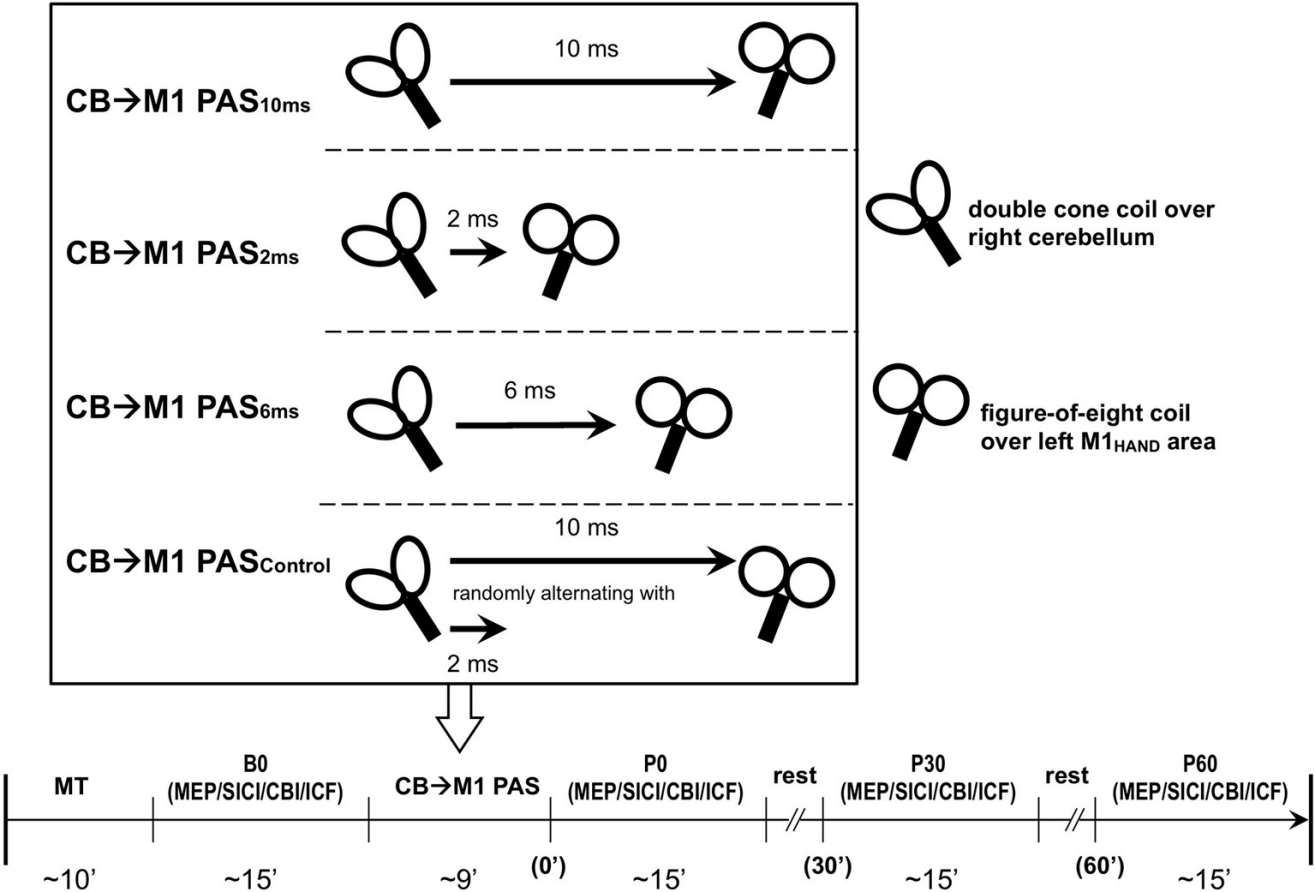
**Experimental design and time line of the cerebellum (CB)→motor cortex (M1) paired associative stimulation (PAS) study.** Four PAS protocols were applied in healthy subjects: CB→M1 PAS_10 ms_ [i.e., interstimulus interval between magnetic pulses to right lateral CB and hand area of left M1 (M1_HAND_) was 10 ms], CB→M1 PAS_6 ms_ (interstimulus interval of 6 ms), CB→M1 PAS_2 ms_ (interstimulus interval of 2 ms), and CB→M1 PAS_Control_ (randomly alternating intervals of 2 and 10 ms). The timeline in the lower part of the diagram shows the order of measurements and their approximate durations (in min) before (B0) and after CB→M1 PAS (P0, P30, and P60: immediately and 30 and 60 min post-PAS). Abbreviations: MT, motor threshold; MEP, motor-evoked potential; SICI, short-interval intracortical inhibition; CBI, cerebellar-motor cortex inhibition; ICF, intracortical facilitation.

Subjects were seated on a comfortable reclining chair with both arms relaxed. Cortical excitability of the hand representation of the left primary motor cortex (M1_HAND_) was tested with single-pulse and paired-pulse TMS in blocks of measurements immediately before CB→M1 PAS (baseline, B0) and immediately, 30 and 60 min after CB→M1 PAS (P0, P30, and P60, respectively). The target muscle for EMG recordings was the first dorsal interosseus (FDI) of the right hand. The individual resting motor threshold (RMT) and active motor threshold (AMT) were determined over the left M1_HAND_, and AMT was, in addition, determined over the inion prior to baseline. Thresholds were determined to the nearest 1% of maximum stimulator output (MSO) using the relative frequency method (Rossini et al., 1994; Groppa et al., 2012). RMT was defined as the lowest intensity that was sufficient to elicit MEPs = 50 μV in at least 5 of 10 consecutive trials. AMT was tested in the slightly activated FDI (~10% of maximum voluntary contraction) and defined as the lowest stimulator intensity to elicit an MEP = 100 μV in the average of five consecutive trials (Ziemann et al., 1996). Left M1_HAND_ excitability measurements at time points B0, P0, P30, and P60 consisted of MEP amplitude, short-interval intracortical inhibition (SICI), CBI and, in a subset of subjects, intracortical facilitation (ICF) as detailed below (Figure 1). All these measures were obtained in the voluntarily relaxed FDI. Maintenance of muscle relaxation was monitored by online audio-visual feedback of high-gain (50 μV/division) EMG.

### Measurement of motor-evoked potential (MEP) amplitude

TMS was delivered through a focal figure-of-eight stimulating coil (inner diameter of each wing, 70 mm) connected via a BiStim module to two Magstim 200 magnetic stimulators (Magstim Co., Carmarthenshire, Wales, UK) with a monophasic current waveform. The coil was held tangential to the scalp over the presumed hand area of the left M1 with the handle pointing backwards and ~45° away from the midline, thus inducing a current in M1 from posterior-lateral to anterior-medial which is optimal for activating corticospinal neurons transsynaptically (Di Lazzaro et al., 2008). The optimal coil position (“hot spot”; M1_HAND_) was determined as the site where TMS at a slightly suprathreshold intensity produced consistently the largest MEPs in the right FDI. This site was marked on the scalp to ensure a constant placement of the coil throughout the session. At B0, the intensity of TMS was adjusted to produce MEPs of on average 1 mV in peak-to-peak amplitude (MEP_1 mV_) in the resting FDI. The same intensity was applied throughout all post cerebellum-to-M1 PAS (CB→M1 PAS) measurements. The MEPs were recorded using Ag-AgCl electrodes with the active electrode mounted on the motor point of the FDI and the reference electrode on the proximal phalanx of the index finger. The raw EMG was bandpass filtered (20 Hz–2 kHz; Counterpoint Mk2 Electromyograph; Dantec, Skovlunde, Denmark), digitized at a rate of 5 kHz (CED Micro 1401; Cambridge Electronic Design, Cambridge, UK) and stored in a laboratory computer for offline analysis (Spike2 for Windows, Version 3.05, CED). Twenty trials of MEPs were obtained at each time point (B0, P0, P30, and P60). For MEP and all other recordings (SICI, ICF, and CBI) the intertrial interval varied randomly ranging from 7.5–12.5 s to limit anticipation of the next trial. Peak-to-peak MEP amplitudes were measured in the single trials, and the mean at each time point was taken as a measure of corticospinal excitability (Ziemann and Hallett, 2007).

### Measurement of short-interval intracortical inhibition (SICI) and intracortical facilitation (ICF)

SICI and ICF were studied using an established paired-pulse TMS protocol (Kujirai et al., 1993; Ziemann et al., 1996). The two magnetic stimuli were given through the same figure-of-eight stimulating coil over the left M1_HAND_ and the effect of the subthreshold conditioning stimulus (CS) on the test MEP elicited by the subsequent suprathreshold test stimulus (TS) was investigated. SICI was assessed at an interstimulus interval (ISI) of 2.0 ms because at this interval SICI is not contaminated by short-interval intracortical facilitation (SICF) (Peurala et al., 2008). At B0, the CS intensity was adjusted to produce approximately 50% inhibition in order to provide highest sensitivity for detection of changes in SICI after CB→M1 PAS. The CS intensities ranged from 70% to 90% AMT in different individuals. This CS intensity was kept constant throughout the experiment. ICF was assessed in a subset of subjects in a separate block of trials at an ISI of 10 ms. The CS intensities ranged from 75% to 95% AMT in different individuals to produce consistent test MEP facilitation as described in previous studies (Ziemann et al., 1996; Di Lazzaro et al., 2006). This CS intensity was kept constant throughout the experiment. The TS intensity was adjusted to elicit MEP_1 mV_ when unconditioned by CS. Whenever CB→M1 PAS resulted in a change in test MEP amplitude, TS intensity was adjusted to provide MEP_1 mV_ throughout all time points of the experiment. This is important because variation of test MEP amplitude influences expression of SICI and ICF (Sanger et al., 2001; Müller-Dahlhaus et al., 2008). Twelve paired CS-TS trials and twelve TS alone trials were recorded in pseudorandomized order. Conditional averages were calculated, and SICI and ICF were expressed by the mean conditioned MEP amplitude as a percentage of the unconditioned mean (Kujirai et al., 1993).

### Measurement of cerebello-motor cortex inhibition (CBI)

**Figure 2:**
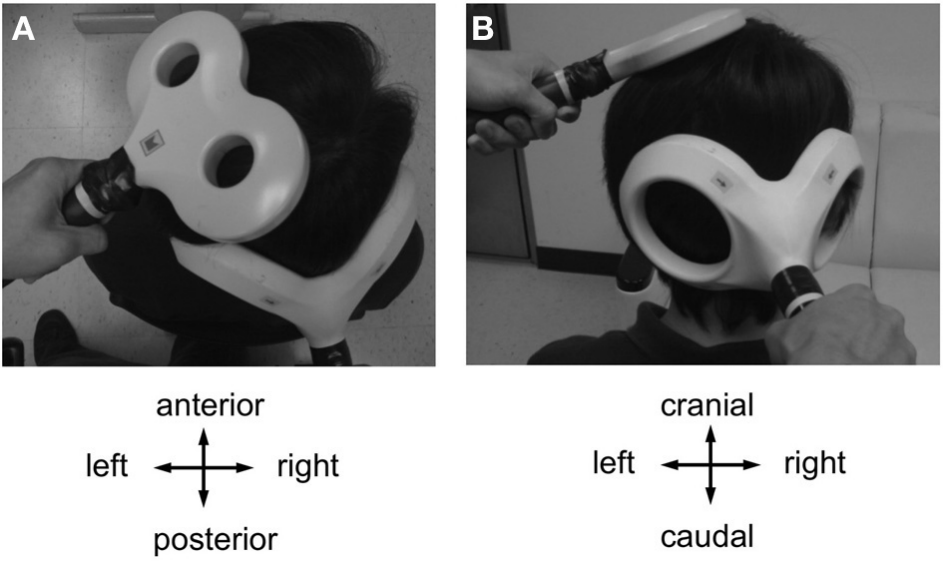
**Coil positions for cerebellar-motor cortical inhibition (CBI) and CB→M1 PAS (A, view from above; B, view from behind).** A double cone coil was placed over the midpoint of the inion and the right mastoid process for stimulation of the right lateral cerebellum. The coil was oriented to induce an upward current in the cerebellum. A figure-of-eight coil was used to stimulate the left M1_HAND_. The coil was oriented to induce a current in M1 directed from posterior-lateral to antero-medial. Motor-evoked potentials were recorded from the first dorsal interosseous muscle of the right hand.

CBI was studied using an established paired-coil protocol (Ugawa et al., 1995). Individual AMT of the descending corticospinal tract was determined by placing a double cone coil (inner diameter of each wing, 110 mm; Magstim Co., UK) over the inion with the coil junction oriented vertically and to induce a downward electric current in the underlying tissue. For the CBI measurements, the center of the coil was moved rightwards off the midpoint by 3 cm along a line between the inion and the right mastoid process, and the coil orientation was rotated by 180° to induce an upward electric current in the right cerebellar hemisphere (Figure 2) (Ugawa et al., 1995; Daskalakis et al., 2004). The conditioning stimulus (CS) was delivered through this coil, and the intensity was set to 95% AMT (as determined over the inion) and kept constant throughout the experiment. This low intensity was chosen to minimize confounding effects due to direct brainstem stimulation (Ugawa et al., 1995; Fisher et al., 2009). The test stimulus (TS) was delivered through a figure-of-eight coil placed over the left M1_HAND_ area and, whenever necessary, its intensity was adjusted throughout the four time points of the experiment (B0, P0, P30, and P60) to elicit an unconditioned test MEP in the right FDI of 0.6–0.8 mV in peak-to-peak amplitude (MEP_0.7 mV_). Compared to the SICI and ICF measurements, a slightly lower test MEP amplitude was chosen because CBI was found to be more significant at this lower amplitude (Ugawa et al., 1995; Pinto and Chen, 2001; Daskalakis et al., 2004). CBI was assessed at an ISI of 7 ms which provided clear MEP inhibition in previous studies (Ugawa et al., 1995; Pinto and Chen, 2001), and is not affected by potential contamination from an MEP inhibition at longer ISIs induced by stimulation of peripheral nerve afferents (Werhahn et al., 1996). Twelve CS-TS trials and twelve TS alone trials alone were recorded in pseudo-randomized order. Conditional averages were calculated, and CBI was expressed by the mean conditioned MEP amplitude as a percentage of the unconditioned mean (Ugawa et al., 1995).

### Cerebellum to motor cortex paired associative stimulation (CB→M1 PAS)

Maximal CBI occurs when TS over M1_HAND_ is given 6–7 ms after a CS over the contralateral cerebellum (Ugawa et al., 1995). Therefore, an ISI of 6–7 ms would result in arrival of the afferent signal elicited by the cerebellar CS at the *same time* in M1_HAND_ when TS of the M1_HAND_ generates actions potentials in excitatory interneurons and corticospinal neurons, ISIs longer than 7 ms would result in arrival of the afferent signal *before* TS-induced actions potentials in M1_HAND_, and this order of events in the M1_HAND_ would be reversed if ISIs shorter than 6 ms were applied. According to other PAS protocols that have paired electrical stimulation of a peripheral nerve (Stefan et al., 2000, 2002; Wolters et al., 2003; Ziemann et al., 2004; Müller-Dahlhaus et al., 2010) or a magnetic CS to the supplementary motor area (Arai et al., 2011) with TMS of M1_HAND_, and according to the principles of spike-timing dependent bidirectional plasticity (Markram et al., 1997; Bi and Poo, 2001) repeated pairing at long intervals (≥6 ms) are expected to result in LTP-like changes, while shorter intervals should lead to LTD-like changes. However, CS results in an inhibitory input to the M1_HAND_, most likely through activation of Purkinje cells, the principal inhibitory neurons of the cerebellar hemispheres (Ugawa et al., 1995). STDP of inhibitory circuits has not been studied well in basic experiments (Lamsa et al., 2010). Therefore, it is difficult to predict the outcome of the present experiments. It might be speculated that LTP-like change of this inhibitory input leads to a long-term MEP decrease (due to strengthened inhibitory control of corticospinal cells), and *vice versa*, LTD-like change to a long-term MEP increase.

In separate sessions, CB→M1 PAS was applied at one of four different ISIs: 10 ms (13 subjects), 6 ms (6 subjects), 2 ms (13 subjects) or trial-by-trial randomly alternating ISIs of 10 ms and 2 ms (9 subjects) (Figure 1). The randomly alternating protocol was developed in our group originally for the conventional PAS protocol (i.e., pairing of electrical peripheral nerve stimulation with TMS of the contralateral M1_HAND_) where it did not induce any significant change of MEP amplitude (Müller et al., 2007; Lu et al., 2009). The intensity of CS was kept at 95% AMT determined over the inion. A total of 120 CS-TS pairs were delivered at a frequency of 0.25 Hz in each of the sessions (i.e., the duration of CB→M1 PAS was 8 min). Several subjects participated in more than one CB→M1 PAS condition. In these cases, the order of CB→M1 PAS conditions was pseudo-randomized and the minimum interval between two successive sessions in a given subject was 5 days in order to avoid interactions between sessions.

### Data analysis and statistics

Statistics were performed with IBM SPSS (Version 20). Data are presented as means ± SEM if not stated otherwise. For all tests a *P* value of < 0.05 was considered significant. Separate mixed repeated measures analyses of variance (rmANOVA) were used to test the effects of CB→M1 PAS on MEP amplitude, SICI, CBI, and ICF. The within subject effect was TIME (B0, P0, P30, and P60), and the between-subject effect was PAS PROTOCOL (CB→M1 PAS_10 ms_, CB→M1 PAS_6 ms_, CB→M1 PAS_2 ms_, and CB→M1 PAS_Control_). Conditional on a significant *F* value, *post-hoc* comparisons were performed using paired-sample *t*-tests with Fisher's LSD correction for multiple comparisons. Violation of sphericity was checked with Mauchly's test and degrees of freedom were adjusted whenever Mauchly's W < 0.05 using the Greenhouse-Geisser correction.

## Results

None of the subjects experienced any noticeable adverse effects during or after the study. All subjects were cooperative throughout the experimental procedures. The grand averages (± SD) across all sessions and subjects were for RMT: 43.4 ± 5.8% MSO; AMT of M1_HAND_: 33.4 ± 4.2% MSO; MEP_0.7 mV_: 49.6 ± 8.2% MSO; MEP_1 mV_: 52.7 ± 8.8% MSO; AMT over inion: 33.7 ± 2.9% MSO. There were no differences between PAS protocols for any of these measures (all *P* > 0.5).

### MEP amplitude

**Figure 3:**
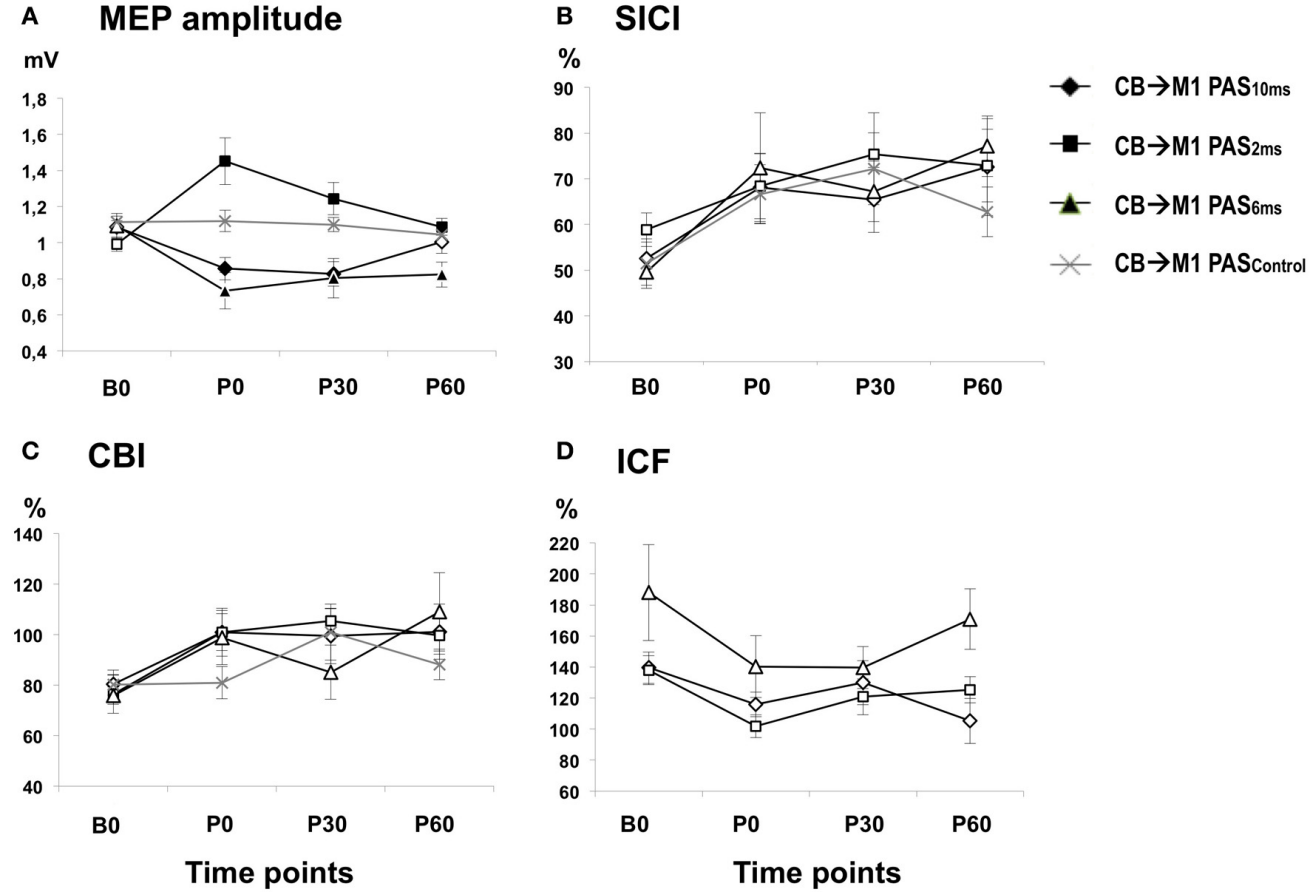
**(A)** Means (± SEM) of MEP amplitude (in mV) at baseline (B0), immediately (P0), 30 min (P30) and 60 min (P60) after CB→M1 PAS (rhomboids: CB→M1 PAS_10 ms_; triangles: CB→M1 PAS_6 ms_; squares: CB→M1 PAS_2 ms_; crosses: CB→M1 PAS_Control_). Filled symbols denote significant differences in MEP amplitude after CB→M1 PAS compared to B0. Note significant MEP suppression at P0 and P30 after CB→M1 PAS_10 ms_ and at P0–P60 after CB→M1 PAS_6 ms_ but MEP potentiation at P0–P60 after CB→M1 PAS_2 ms_. In contrast, MEP amplitude remained unchanged after CB→M1 PAS_Control_. **(B,C)** Mean SICI and CBI (given as percentage of the conditioned MEP/unconditioned MEP). Otherwise, same conventions as in **(A)**. SICI and CBI decreased significantly but non-specifically across all three PAS conditions, but this effect was no longer significant at the level of single time points and PAS conditions in the *post-hoc* tests. **(D)** Mean ICF (given as percentage of the conditioned MEP/unconditioned MEP). Same conventions as in **(A)**. CB→M1 PAS had no significant effect on ICF.

There was a main effect of PAS PROTOCOL [*F*_(3, 111)_ = 6.97, *P* = 0.001] and a significant PAS PROTOCOL × TIME interaction [*F*_(9, 333)_ = 7.94, *P* < 0.001] (Table 1, Figure 3A). *Post-hoc* comparisons showed significant differences of CB→M1 PAS_10 ms_ vs. CB→M1 PAS_2 ms_, CB→M1 PAS_6 ms_ vs. CB→M1 PAS_2 ms_, and CB→M1 PAS_6 ms_ vs. CB→M1 PAS_Control_ (all *P* < 0.05).

**Table 1:** **Mixed rmANOVA of the CB→M1 PAS effects on MEP, SICI, and CBI**.

		**MEP**		**SICI**		**CBI**	
	**df**	***F***	***P***	***F***	***P***	***F***	***P***
PAS PROTOCOL	3,37	**6.97**	**0.001**	0.22	0.88	0.83	0.49
TIME	3,111	1.63	0.19	**6.33**	**0.001**	**4.08**	**0.009**
PAS PROTOCOL × TIME	9,111	**7.94**	**< 0.001**	0.38	0.94	0.18	0.91

One-way rmANOVA of CB→M1 PAS_10 ms_ revealed a significant effect of TIME [*F*_(3, 36)_ = 7.21, *P* = 0.001], which was explained by significant MEP amplitude depression at P0 and P30 when compared to B0 (mean MEP amplitudes at B0: 1.09 ± 0.04 mV, P0: 0.86 ± 0.06 mV, P30: 0.83 ± 0.07 mV, both *P* < 0.01) but no longer at 60 min (P60) (1.00 ± 0.06 mV, *P* = 0.11; diamonds in Figure 3A). CB→M1 PAS_6 ms_ also showed a significant effect of TIME [*F*_(3, 15)_ = 6.46, *P* = 0.005], which was explained by significant MEP amplitude depression at P0, P30, and P60 when compared to B0 (mean MEP amplitudes at B0: 1.09 ± 0.06 mV, P0: 0.73 ± 0.10 mV, P30: 0.80 ± 0.11 mV, P60: 0.82 ± 0.07 mV, all *P* < 0.05; triangles in Figure 3A). CB→M1 PAS_2 ms_ revealed a significant effect of TIME [*F*_(3, 36)_ = 8.61, *P* < 0.001], which was explained by significant MEP amplitude potentiation at P0, P30, and P60 when compared to B0 (mean MEP amplitudes at B0: 0.99 ± 0.04 mV, P0: 1.45 ± 0.13 mV, P30: 1.24 ± 0.09 mV, P60: 1.09 ± 0.05 mV, all *P* < 0.05; squares in Figure 3A). Finally, CB→M1 PAS_Control_ showed no effect of TIME [*F*_(3, 24)_ = 0.75, *P* = 0.54; crosses in Figure 3A].

There were no differences of mean MEP amplitudes at B0 across CB→M1 PAS protocols (CB→M1 PAS_10 ms_: 1.09 ± 0.04 mV; CB→M1 PAS_6 ms_: 1.09 ± 0.07 mV; CB→M1 PAS_6 ms_: 0.99 ± 0.04 mV; CB→M1 PAS_Control_: 1.11 ± 0.04 mV; all *P* > 0.05) that could have accounted for the differential effects of the CB→M1 PAS protocols on MEP amplitude.

### SICI

There was a main effect of TIME [*F*_(3, 111)_ = 6.33, *P* = 0.001] but no significant effects of PAS PROTOCOL or of the TIME × PAS PROTOCOL interaction (both *P* > 0.8) (Table 1). The effect of TIME was explained by a non-specific decrease of SICI at all post CB→M1 PAS time points compared to baseline (B0: 53.9 ± 2.2%, P0: 68.5 ± 3.9%, P30: 70.3 ± 4.2%, P60: 71.2 ± 4.5%; all *P* < 0.005; Figure 3B). However, this effect of TIME was weak and no longer significant when tested separately for the different PAS protocols (all *P* > 0.05). SICI at time point B0 did not differ between PAS protocols (CB→M1 PAS_10 ms_: 52.6 ± 4.3%; CB→M1 PAS_6 ms_: 49.6 ± 3.6%; CB→M1 PAS_2 ms_: 58.8 ± 3.7%; CB→M1 PAS_Control_: 51.4 ± 5.7%; all *P* > 0.15, Figure 3B). There were no effects of TIME, PAS PROTOCOL or of the TIME × PAS PROTOCOL interaction on unconditioned test MEP amplitude (all *P* > 0.2), indicating that maintenance of MEP_1 mV_ across time points and PAS protocols (grand average, 0.95 ± 0.23 mV) was successfully achieved during the SICI measurements.

### CBI

There was a main effect of TIME [*F*_(3, 111)_ = 4.08, *P* = 0.009] but no effects of PAS PROTOCOL or of the TIME × PAS PROTOCOL interaction on CBI (both *P* > 0.4) (Table 1). The effect of TIME was explained by a non-specific decrease of CBI at all post CB→M1 PAS time points compared to baseline (B0: 78.4 ± 3.1%, P0: 96.3 ± 4.5%, P30: 99.6 ± 5.0%, P60: 99.0 ± 4.9%; all *P* < 0.01; Figure 3C). However, this effect of TIME was weak and no longer significant when tested separately for the different PAS protocols (all *P* > 0.05). CBI at time point B0 did not differ between PAS protocols (CB→M1 PAS_10 ms_: 80.4 ± 5.6%; CB→M1 PAS_6 ms_: 75.8 ± 3.4%; CB→M1 PAS_2 ms_: 76.5 ± 7.6%; CB→M1 PAS_Control_: 80.1 ± 5.0%; all *P* > 0.5, Figure 3C). There were no effects of TIME, PAS PROTOCOL or the TIME × PAS PROTOCOL interaction on unconditioned test MEP amplitude (all *P* > 0.05), indicating that maintenance of MEP_0.7 mV_ across time points and PAS protocols (grand average: 0.81 ± 0.24 mV) was successfully achieved during the CBI measurements.

### ICF

There were no effects of TIME [*F*_(3, 33)_ = 2.36, *P* = 0.08], PAS PROTOCOL [*F*_(2, 11)_ = 4.01, *P* = 0.06] or of the TIME × PAS PROTOCOL interaction [*F*_(6, 33)_ = 0.63, *P* = 0.70] on ICF (Figure 3D). ICF at time point B0 did not differ between PAS Protocols (all *P* > 0.2, Figure 3D). There were no effects of TIME, PAS PROTOCOL or the TIME × PAS PROTOCOL interaction on unconditioned test MEP amplitude (all *P* > 0.05), indicating that maintenance of MEP_1 mV_ across time points and PAS protocols (grand average: 0.96 ± 0.26 mV) was successfully achieved during the ICF measurements.

## Discussion

This study demonstrates, to the best of our knowledge for the first time, bidirectional long-term (> 30 min) STDP-like plasticity of MEP amplitude induced by associative stimulation of the cerebello-dentato-thalamo-M1 pathway and the corticospinal output network in M1: CB→M1 PAS_2 ms_ resulted in an increase in corticospinal excitability indexed by MEP amplitude, while CB→M1 PAS_6 ms_ and CB→M1 PAS_10 ms_ resulted in a MEP decrease, and CB→M1 PAS_Control_ in no change. The data extend previous studies showing bidirectional STDP-like plasticity in M1 when focal TMS of the M1_HAND_ was paired with conditioning stimulation of a peripheral nerve (Stefan et al., 2000; Wolters et al., 2003) or with another TMS pulse applied to the ipsilateral ventral premotor cortex (Buch et al., 2011), suggesting that STDP-like plasticity in human M1 is a generally operating principle for various afferent inputs.

The “classical” PAS protocol to induce STDP-like plasticity employs repeated pairing of electrical stimulation of the median nerve with focal TMS of the contralateral M1_HAND_ (Stefan et al., 2000; Wolters et al., 2003; Ziemann et al., 2004; Müller et al., 2007) (for review, Müller-Dahlhaus et al., 2010). The ISI between the peripheral electrical stimulus and TMS of M1_HAND_ is critical for the expression of LTP-like vs. LTD-like changes in MEP amplitude: if the ISI is equal or longer than the individual latency of the earliest cortical component (N20) of the median nerve somatosensory-evoked potential, then a long-lasting (typically > 30 min) LTP-like increase in MEP amplitude occurs in the majority of subjects. In contrast, ISIs that is shorter by 5–15 ms than the N20 latency result in a LTD-like MEP decrease (Müller-Dahlhaus et al., 2010) If the afferent volley from peripheral nerve stimulation produced excitatory postsynaptic potentials (EPSPs) in the motor cortical output neurons then these EPSPs would precede action potential generation in corticospinal cells by TMS with the longer ISIs, while the order of these events would be reversed with the shorter ISIs. The resulting bidirectional M1 LTP/D-like plasticity is strongly reminiscent to STDP studied in neuronal slices or cultures where the temporal order of repeated pairing of EPSPs and action potentials determines the direction of synaptic plasticity (Markram et al., 1997; Bi and Poo, 2001; Dan and Poo, 2004). However, one critical issue with the classical PAS protocol is that the afferent electrical stimulation to the peripheral nerve causes MEP inhibition at intervals that are typically associated with LTP-like plasticity. This MEP inhibition has been termed short-latency afferent inhibition (SAI) and is mediated by cortical inhibitory interneurons (Tokimura et al., 2000; Di Lazzaro et al., 2007). It was also demonstrated that the presence of SAI causes a short-lasting decrease of SICI (Stefan et al., 2002; Alle et al., 2009). Since the level of local inhibition exerts a powerful role in regulating synaptic plasticity in M1 (Castro-Alamancos et al., 1995; Hess et al., 1996; Ziemann et al., 1998; Heidegger et al., 2010), this disinhibition (i.e., the decrease of SICI) may be crucial for an LTP-like effect to occur in the presence of SAI (Stefan et al., 2002). Another recently established PAS protocol is applied to the interhemispheric connection between the two M1 (Koganemaru et al., 2009; Rizzo et al., 2009). An ISI of 8 ms between TMS of the conditioning left M1_HAND_ and TMS of the conditioned right M1_HAND_ results in a LTP-like MEP increase in the right M1_HAND_ and a concomitant (but not correlated) decrease of short-latency interhemispheric inhibition (SIHI) (Rizzo et al., 2009). Similar to the classical PAS protocol, the paradox is that the LTP-like effect occurs although the conditioning M1 stimulation causes MEP inhibition in the conditioned M1_HAND_ in the form of SIHI (Ferbert et al., 1992; Di Lazzaro et al., 1999). However, triple-pulse TMS experiments revealed that the presence of SIHI reduces SICI (Daskalakis et al., 2002; Müller-Dahlhaus et al., 2008). Again, this disinhibition might be critical to permit the LTP-like effect to occur in this protocol.

Similar to those studies, CB conditioning stimulation also decreases MEP amplitude in the contralateral M1_HAND_, termed cerebellar-motor inhibition (CBI) (Ugawa et al., 1995; Werhahn et al., 1996), and the presence of CBI reduces SICI (Daskalakis et al., 2004). However, in contrast to the classical and interhemispheric PAS protocols, CB→M1 PAS_10 ms_ and CB→M1 PAS_6 ms_ resulted in LTD-like MEP amplitude decrease although the CB conditioning effect reached M1 at the same time or prior to M1_HAND_ stimulation, given a CB→M1 conduction time of 5–6 ms (Ugawa et al., 1995; Werhahn et al., 1996; Pinto and Chen, 2001).

We can currently only speculate about the reasons for this apparent discrepancy. One important difference of CB→M1 PAS compared to the classical and interhemispheric PAS protocols is that TMS over the lateral cerebellum likely activates Purkinje cells, i.e., the major inhibitory interneurons of the cerebellum (Ugawa et al., 1995). In contrast, the conditioning pulses in the other PAS protocols activate primarily excitatory pathways, namely mixed or cutaneous nerve fibers in the classical PAS protocol (Tokimura et al., 2000) and glutamatergic interhemispheric fibers in the interhemispheric PAS protocol (Ferbert et al., 1992) that project onto inhibitory interneurons within M1. The Purkinje cells inhibit deep cerebellar nuclei, which facilitate tonically the contralateral M1 through the dentato-thalamo-M1 pathway (Allen and Tsukahara, 1974; Thach, 1987; Middleton and Strick, 1998). Therefore, activation of the Purkinje cells leads to M1 disfacilitation. Repeated TMS of M1 at a time when CB conditioning stimulation has inhibited this tonically active pathway should lead to Hebbian LTD-like MEP decrease, similar to LTD induced in hippocampal slices when a high-frequency conditioning input was negatively correlated in time with a test input (Stanton and Sejnowski, 1989). The LTP-like MEP increase after CB→M1 PAS_2 ms_ implies a reversal of the order of these events in M1, i.e., action potential generation in M1 corticospinal cells regularly preceded the disfacilitation of the dentato-thalamo-M1 projection. This may have caused a transient strengthening of this tonic input because it was active above average at the time of TMS-induced action potential generation.

Our data are compatible with two 1 Hz repetitive TMS (rTMS) studies of the lateral cerebellum, which demonstrated an increase in MEP amplitude (Oliveri et al., 2005; Fierro et al., 2007). Low-frequency rTMS leads to depression of excitability of the stimulated brain area (Chen et al., 1997; Ziemann et al., 2008). Therefore, the putative depression of Purkinje cell excitability would lead to reduced inhibitory regulation of the dentate-thalamo-M1 pathway and consequently to increased tonic excitatory input to M1.

At a first glance, it might be surprising that CBI did not change accordingly, i.e., decreased after CB→M1 PAS_10 ms_ and CB→M1 PAS_6 ms_ and increased after CB→M1 PAS_2 ms_. This lack of change in the strength of the stimulated conditioning pathway is very similar to classical PAS where SAI representing motor cortical inhibition mediated by an ascending central cholinergic projection (Di Lazzaro et al., 2000) does not change (Stefan et al., 2002; Hamada et al., 2012).

Other recent studies demonstrated a significant bidirectional change of CBI after anodal (CBI increase) vs. cathodal transcranial direct current stimulation (CBI decrease) of the lateral cerebellum (Galea et al., 2009), or a reduction of CBI after 1 Hz rTMS or continuous theta-burst stimulation (Popa et al., 2009) without changes in MEP amplitude. Together, these findings indicate that the modifications of M1 excitability (indexed by MEP amplitude) and CBI are often dissociated.

The non-specific decrease in SICI observed after all four PAS protocols (cf. Figure 3B) was unrelated to the bidirectional modification of MEP amplitude. SICI remained unaffected by 1 Hz rTMS of the lateral cerebellum (Oliveri et al., 2005; Fierro et al., 2007). Thus, it is unlikely that the low-frequency (0.25 Hz) stimulation of the cerebellum *per se* employed in all three PAS protocols of our study was responsible for the SICI change. One possible explanation is provided by the observation that STDP protocols in rat somatosensory cortex resulted always in LTD at excitatory synapses from pyramidal cells onto fast-spiking inhibitory interneurons, irrespective of the interval between the pre- and postsynaptic spikes (Lu et al., 2007). Fast-spiking interneurons are the parvalbumin positive basket cells and chandelier cells (Kawaguchi and Kondo, 2002), which are currently thought to mediate SICI (Ilic et al., 2002; Di Lazzaro et al., 2007).

CB→M1 PAS did not affect ICF in a STDP-like manner (Figure 3D). Previous studies on modulation of ICF by CB stimulation showed inconsistent results. One Hz rTMS resulted either in an ICF increase (Oliveri et al., 2005) or an ICF decrease (Fierro et al., 2007), with a concomitant increase in MEP amplitude (Fierro et al., 2007) or the problem that the observed changes in ICF were not controlled for increases in test MEP amplitude (Oliveri et al., 2005). Continuous theta-burst stimulation of the lateral cerebellum resulted in MEP amplitude decrease but no change in ICF, while intermittent theta-burst stimulation led to an increase in MEP amplitude but a decrease in ICF (Koch et al., 2008). In summary, this corroborates the notion of dissociable physiological mechanisms that underlie MEP amplitude vs. ICF (Di Lazzaro et al., 2006, 2011; Ni et al., 2011). As with the classical PAS protocol (Stefan et al., 2000; Müller-Dahlhaus et al., 2010) the present findings suggest that MEP amplitude is the most suitable marker to demonstrate STDP-like plasticity. This is most likely explained by the fact that MEP amplitude *directly* tests synaptic excitability of exactly those excitatory connections onto the corticospinal pathway (Hallett, 2007; Di Lazzaro et al., 2008), which are stimulated during PAS and undergo change in synaptic strength in accord with the principles of STDP.

It is a long-held concern that the magnetic stimulation of the lateral cerebellum with a large double cone coil (Figure 2) is non-specific and may, in addition, excite sensory afferent fibers in the brachial plexus or the spinal dorsal nerve roots (Werhahn et al., 1996). This is, however, very unlikely to explain the present results because excitation of fast-conducting somatosensory afferents should behave like classical PAS (Stefan et al., 2000; Wolters et al., 2003), i.e., the CB→M1 PAS_10 ms_ and CB→M1 PAS_6 ms_ protocols should have resulted in an LTP-like increase of MEP amplitude and the CB→M1 PAS_2 ms_ in a LTD-like MEP decrease, while we observed MEP modifications in the opposite directions.

Another concern is direct excitation of the pyramidal tract at the level of the foramen magnum (Ugawa et al., 1994; Fisher et al., 2009) and antidromic propagation of the elicited action potentials via recurrent collaterals into the M1 circuitry (Stefanis and Jasper, 1964). Again, this is a highly unlikely scenario for the following reasons. The threshold was determined with conditions which typically result in lowest threshold values, i.e., with the coil centered over the midline at the inion level and with a downward directed induced current (Ugawa et al., 1994). For stimulation of the lateral cerebellum, a subthreshold stimulus intensity of 95% AMT was used, the coil was lateralized by 3 cm and an upward directed induced current was applied. These are the optimal conditions for eliciting CBI in the absence of direct stimulation of the corticospinal tract (Ugawa et al., 1995; Pinto and Chen, 2001; Fisher et al., 2009).

In summary, our findings suggest the possibility to induce STDP-like plasticity in human M1 by PAS of the contralateral cerebellum and M1. This bidirectional modification of M1 excitability may prove useful for correcting abnormal M1 excitability caused by cerebellar disease. Future studies may investigate the behavioral significance of this plasticity, in particular with respect to motor performance and motor adaptation.

### Conflict of interest statement

The authors declare that the research was conducted in the absence of any commercial or financial relationships that could be construed as a potential conflict of interest.
